# KIAA1217 Promotes Epithelial-Mesenchymal Transition and Hepatocellular Carcinoma Metastasis by Interacting with and Activating STAT3

**DOI:** 10.3390/ijms23010104

**Published:** 2021-12-22

**Authors:** Yanhong Wang, Na Li, Yanping Zheng, Anqing Wang, Chunlei Yu, Zhenbo Song, Shuyue Wang, Ying Sun, Lihua Zheng, Guannan Wang, Lei Liu, Jingwen Yi, Yanxin Huang, Muqing Zhang, Yongli Bao, Luguo Sun

**Affiliations:** 1 National Engineering Laboratory for Druggable Gene and Protein Screening, Northeast Normal University, Changchun 130024, China; wangyh137@nenu.edu.cn (Y.W.); lin959@nenu.edu.cn (N.L.); zhengyp010@nenu.edu.cn (Y.Z.); wangaq426@nenu.edu.cn (A.W.); yucl885@nenu.edu.cn (C.Y.); suny040@nenu.edu.cn (Y.S.); huangyx356@nenu.edu.cn (Y.H.); baoyl800@nenu.edu.cn (Y.B.); 2 NMPA Key Laboratory for Quality of Cell and Gene Therapy Medicinal Products, Northeast Normal University, Changchun 130024, China; songzb484@nenu.edu.cn (Z.S.); wangsy171@nenu.edu.cn (S.W.); zhenglh015@nenu.edu.cn (L.Z.); wanggn258@nenu.edu.cn (G.W.); liul905@nenu.edu.cn (L.L.); yijw894@nenu.edu.cn (J.Y.); 3 School of Molecular and Cellular Biology, University of Illinois Urbana Champaign, Urbana, IL 61801, USA; zhangmuqing41@gmail.com

**Keywords:** KIAA1217, hepatocellular carcinoma, epithelial-mesenchymal transition, metastasis, STAT3

## Abstract

The survival and prognosis of hepatocellular carcinoma (HCC) are poor, mainly due to metastasis. Therefore, insights into the molecular mechanisms underlying HCC invasion and metastasis are urgently needed to develop a more effective antimetastatic therapy. Here, we report that KIAA1217, a functionally unknown macromolecular protein, plays a crucial role in HCC metastasis. KIAA1217 expression was frequently upregulated in HCC cell lines and tissues, and high KIAA1217 expression was closely associated with shorter survival of patients with HCC. Overexpression and knockdown experiments revealed that KIAA1217 significantly promoted cell migration and invasion by inducing epithelial-mesenchymal transition (EMT) in vitro. Consistently, HCC cells overexpressing KIAA1217 exhibited markedly enhanced lung metastasis in vivo. Mechanistically, KIAA1217 enhanced EMT and accordingly promoted HCC metastasis by interacting with and activating JAK1/2 and STAT3. Interestingly, KIAA1217-activated p-STAT3 was retained in the cytoplasm instead of translocating into the nucleus, where p-STAT3 subsequently activated the Notch and Wnt/β-catenin pathways to facilitate EMT induction and HCC metastasis. Collectively, KIAA1217 may function as an adaptor protein or scaffold protein in the cytoplasm and coordinate multiple pathways to promote EMT-induced HCC metastasis, indicating its potential as a therapeutic target for curbing HCC metastasis.

## 1. Introduction

Cancer is a disease that seriously threatens human life and health worldwide [1]. The occurrence and development of cancer is a complex multistep process involving the accumulation of multiple genetic and epigenetic changes that lead to alterations in a large number of tumor-related genes, such as the activation of oncogenes and the inactivation of tumor suppressor genes [2,3]. However, cancer is still insufficiently understood due to its complexity, which hinders efforts to achieve a complete cure of cancer. Therefore, further explorations of the molecular mechanism underlying the occurrence and development of cancer, such as discovering the role of more tumor-related genes in the progression of cancer, are still indispensable.

KIAA1217, the human homolog of murine Skt (sickle tail), was identified in the large HUGE (human unidentified gene-encoded) protein database [4] and is a macromolecular protein with an unknown function. To date, only a few articles have reported KIAA1217 [5,6]. It is ubiquitously expressed in the cytoplasm [5]. Because it is specifically expressed in the nucleus pulposus of intervertebral discs in humans and mice, KIAA1217 is presumed to be necessary for the normal development of intervertebral discs and is a good candidate gene for lumbar disc herniations in humans [6]. In particular, a novel KIAA1217-RET fusion gene was identified in lung adenocarcinomas [5]. This fusion gene, resulting from the rearrangement of chromosome 10, is produced by the fusion of KIAA1217 (NM_001282767.1) exons 1–11 and RET exons 11–20 in a patient with non-small cell lung cancer [5]. The expression of the KIAA1217-RET fusion gene increases cell proliferation and invasion through the activation of the PI3K/AKT and ERK signaling pathways and ultimately leads to the oncogenic transformation of lung cells [5]. However, the potential role and mechanism of KIAA1217 in the progression of cancer remains unclear.

Liver cancer, of which hepatocellular carcinoma (HCC) accounts for 75–85% of cases, is the sixth most commonly diagnosed cancer (4.7% of the total cases) and the third leading cause of cancer-related death (8.3%) [1]. In particular, HCC is one of the malignant tumors with a high incidence and the worst survival rate in China [7]. In recent years, with the continuous development of HCC diagnostic and treatment methods, the overall survival rate has been improved to a large extent [8,9,10]. However, the prognosis of patients with HCC is still unfavorable, with only an 18% 5-year survival rate from 2009–2015 [11]. This low survival rate is largely attributed to insidious onset, rapid progression, strong infiltration, and early metastasis of HCC [12,13]. Indeed, most patients with liver cancer are diagnosed at an advanced stage, and many have already developed intrahepatic metastasis or even systemic metastases in other tissues [8,9,10,14,15]. Thus, metastasis is one of the key factors affecting the survival and prognosis of patients with HCC [12,13], and the identification of potential key targets and elucidation of the molecular mechanism of HCC metastasis is very important.

Notably, epithelial-mesenchymal transition (EMT) plays a critical role in the invasion and metastasis cascades of cancer [16,17,18], including HCC [19,20,21]. EMT is a reversible biological process in which epithelial cells transform into mesenchymal cell phenotypes under certain conditions [16]. Cancer cells undergoing EMT lose cell polarity and cell-cell adhesions, reorganize the cytoskeleton, downregulate epithelial markers, and upregulate mesenchymal markers [16]. These changes lead to the increased migration and invasion of cancer cells [22]. Studies have shown that EMT is precisely regulated by a complex and dynamic mechanism involving the regulation of multiple signaling pathways and multiple factors, including transcription factors, microRNAs, and growth factors [23,24]. Hence, the mechanism of EMT induction in the invasion and metastasis of HCC must be further explored.

In this study, we found that KIAA1217 expression was frequently upregulated in HCC cell lines and tissues, and high KIAA1217 expression was closely associated with shorter survival of patients with HCC. Then, we described a critical role for KIAA1217 in EMT induction and HCC metastasis and elucidated the underlying molecular mechanisms. We identified KIAA1217 as an oncogenic protein during HCC progression for the first time and suggested it as a potential antimetastatic target for HCC treatment.

## 2. Results

### 2.1. KIAA1217 Is Frequently Upregulated in HCC Tissues and Indicates Shorter Survival

**Figure 1:**
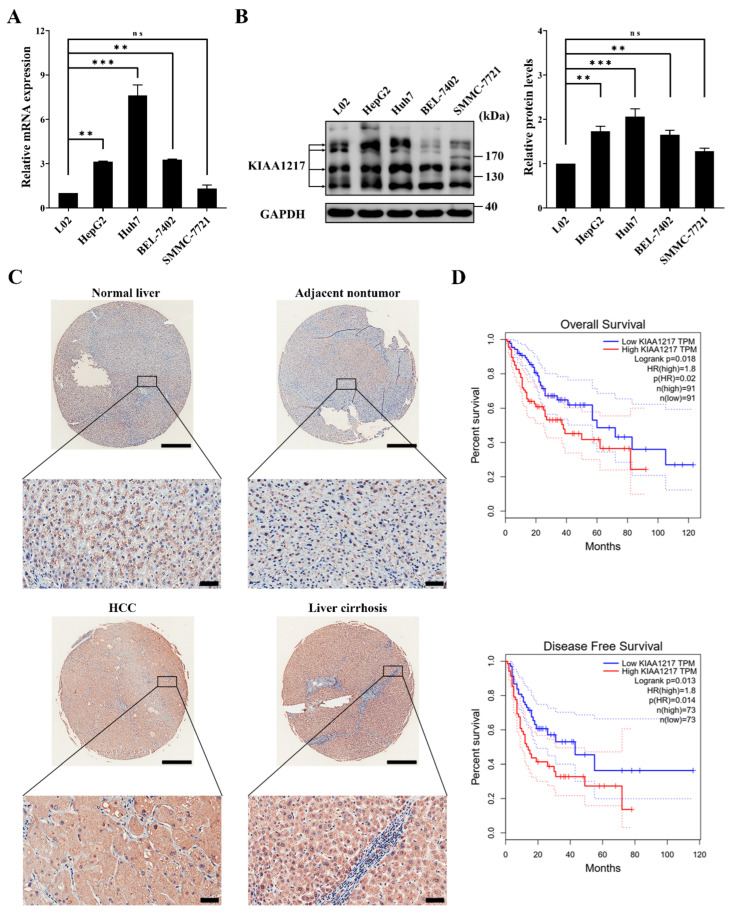
Upregulation of KIAA1217 was frequently observed in HCC samples and indicated shorter survival. (**A**) RT–qPCR analysis of KIAA1217 mRNA expression in HCC cell lines. β-Actin was used as an internal control. (**B**) Left panel, Western blot analysis of KIAA1217 protein expression in HCC cell lines; right panel, quantitative evaluation of the Western blot results. GAPDH served as a loading control. (**C**) Representative images of IHC staining for KIAA1217 expression in normal liver tissue and matched adjacent nontumor tissue, HCC tissue, and cirrhotic liver tissue. Magnification, ×20 (upper panel) and ×200 (lower panel); scale bars: 500 μm (upper panel) and 50 μm (lower panel). (**D**) Kaplan–Meier analysis of the correlation between KIAA1217 expression and the overall survival or disease-free survival of patients with HCC using the GEPIA web server [25]. ns, not significant; ** *p* < 0.01; *** *p* < 0.001. Error bars: SEM.

**Figure 2:**
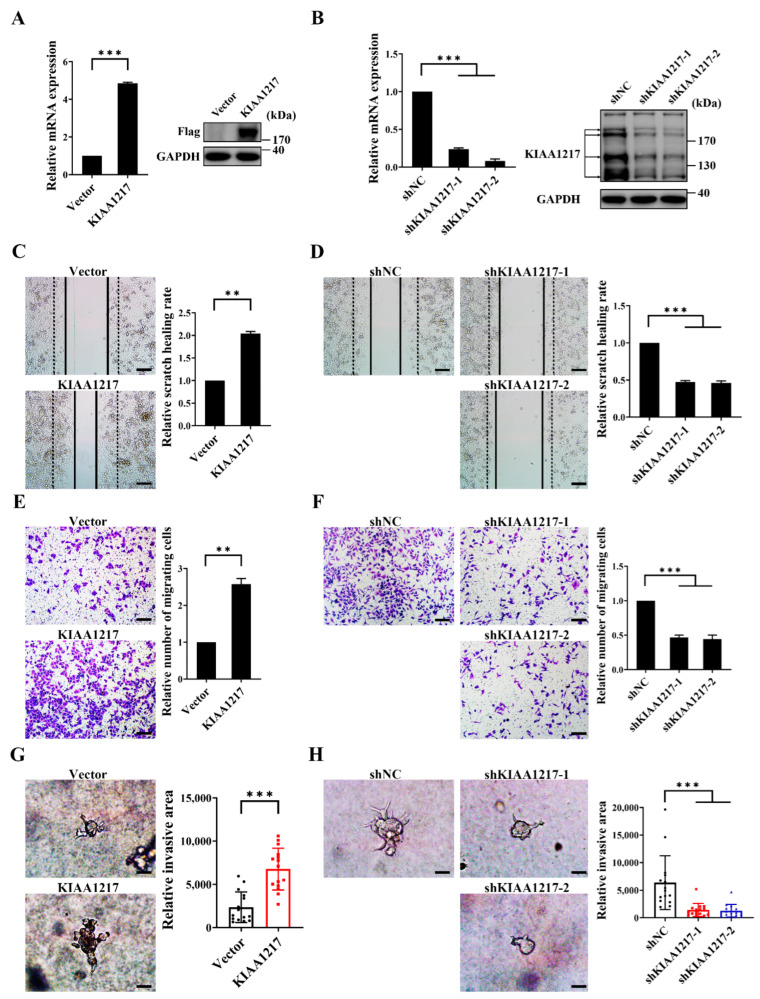
KIAA1217 promoted cell migration and invasion in vitro. (**A**,**B**) The overexpression (**A**) or silencing (**B**) of KIAA1217 in HepG2 cells were confirmed using RT–qPCR and Western blot analyses. β-Actin and GAPDH were used as internal controls for RT–qPCR and Western blot, respectively. (**C**,**D**) Wound-healing assays were performed to detect the migration of HepG2 cells with KIAA1217 overexpression (**C**) or knockdown (**D**) at 48 h after scratching. Magnification ×100; scale bars: 100 μm. (**E**,**F**) Transwell migration assays were performed to detect the migration of HepG2 cells with KIAA1217 overexpression (**E**) or silencing (**F**). Magnification ×100; scale bars: 100 μm. (**G**,**H**) Cancer cell spheroid invasion assays in a 3D setting were performed to detect the invasion of HepG2 cells with KIAA1217 overexpression (**G**) or knockdown (**H**). Magnification ×200; scale bars: 5 μm. ** *p* < 0.01; *** *p* < 0.001. Error bars: SEM.

We first analyzed KIAA1217 expression in HCC cell lines and tissues to investigate its potential role in HCC progression. RT–qPCR (Figure 1A) and Western blot (Figure 1B) analyses of HCC cell lines showed that except for SMMC-7721 cells, KIAA1217 expression was frequently upregulated in the tested HCC cell lines compared with the human immortalized liver cell line L02. Notably, multiple protein bands were detected for KIAA1217, as shown in the Western blot results (Figure 1B). According to the NCBI database, seven splicing variants have been identified, which are named KIAA1217-V1/2/4/5/6/7/8 (Figure S1). Therefore, we postulated that most of the bands shown in Western blot are KIAA1217 variants, which was later confirmed in cells stably transfected with the shRNA since most bands were downregulated by shRNAs targeting KIAA1217 (Figure 2B).

Next, the expression of total KIAA1217 in HCC tissues was detected by performing IHC staining of the TMA slide. As shown in Figure 1C, KIAA1217 was primarily expressed in the cytoplasm, as described previously [5], and KIAA1217 expression was upregulated in some HCC tissues compared with adjacent nontumor tissues and normal liver tissues. Notably, the high expression level of KIAA1217 in patients with liver cirrhosis (83.33%) was significantly higher than that in HCC tissues (47.06%) (Figure 1C). As liver cirrhosis is closely related to the development of HCC [26,27], we considered that the presence of cirrhotic liver tissues interferes with our judgment of the expression of KIAA1217 in HCC tissues compared with adjacent nontumor tissues. However, we still claim that KIAA1217 is expressed at high levels in at least a portion of patients with HCC.

The correlation of KIAA1217 expression with the survival of patients with HCC was also evaluated. The RNA sequencing expression data of HCC samples from TCGA and GTEx databases were used to assess the effect of KIAA1217 expression on the survival of patients with HCC using the Kaplan–Meier method, which was analyzed using the GEPIA web server [25]. As shown in Figure 1D, patients with HCC presenting high KIAA1217 expression had shorter overall survival (OS) and disease-free survival (DFS) times than patients with low expression.

Taken together, the aforementioned data indicate that KIAA1217 is frequently upregulated in HCC cell lines and tissues, and its high expression predicts shorter survival of patients with HCC.

### 2.2. KIAA1217 Promotes HCC Cell Migration and Invasion In Vitro and Lung Metastasis In Vivo

HepG2 cell lines with stable overexpression or knockdown of KIAA1217 were established by transfecting KIAA1217 expression plasmids or lentivirus-mediated shRNA transduction followed by antibiotic selection to investigate the functions of KIAA1217 in HCC. Two targeting shRNAs (shKIAA1217-1 and shKIAA1217-2) were used to establish two HepG2 cell lines with KIAA1217 knockdown. The stable overexpression (Figure 2A) or knockdown (Figure 2B) of KIAA1217 in HepG2 cell lines was confirmed by performing RT–qPCR and Western blot analyses, respectively. Thereafter, we first tested the effect of KIAA1217 on HCC cell proliferation. However, the MTT assay, BrdU incorporation assay, and colony formation assay showed that neither overexpression nor knockdown of KIAA1217 substantially affected HCC proliferation (Figure S2), indicating that KIAA1217 is not involved in regulating HCC proliferation.

Then, we examined the role of KIAA1217 in HCC invasion and metastasis. The wound-healing assay showed that KIAA1217 overexpression accelerated scratch wound-healing by cultured HepG2 cells compared with the vector control (Figure 2C and Figure S3A,C). In contrast, KIAA1217 knockdown delayed scratch healing by HepG2 cells compared with the shNC control (Figure 2D and Figure S3B,D). Similarly, the Transwell migration assay indicated greater numbers of migrating HepG2 cells overexpressing KIAA1217 than control cells (Figure 2E). Conversely, fewer HepG2 cells with KIAA1217 silencing migrated (Figure 2F). The movement of cells in a 3D matrix is more representative of the actual behavior of cells in vivo; thus, we performed a cancer cell spheroid invasion assay in a 3D setting to evaluate the cell invasion capacity and better simulate the environment in vivo. As shown in Figure 2G, spheroids formed by HepG2 cells overexpressing KIAA1217 displayed a larger invasive area than control cells. Instead, spheroids formed by HepG2 cells with KIAA1217 knockdown showed a smaller invasion area than control cells (Figure 2H). In summary, these data indicate that KIAA1217 substantially enhances cell migration and invasion in vitro.

**Figure 3:**
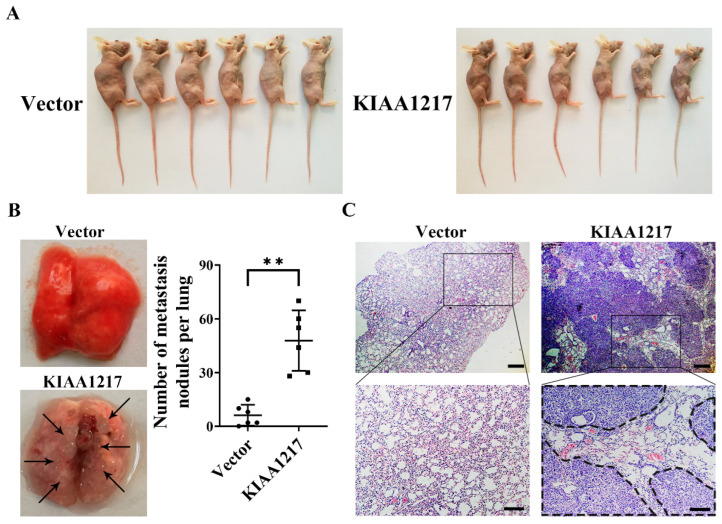
KIAA1217 promoted lung metastasis in vivo. HepG2 cells stably overexpressing KIAA1217 or the vector control were injected intravenously into the tail vein of nude mice. (**A**) Representative images of the physical appearance of nude mice in each group. (**B**) Representative pictures of lungs derived from nude mice in each group and the number of metastatic nodules on the surface of the lungs from each group. ** *p* < 0.01. Error bars: SEM. (**C**) Representative images of H&E staining of lung tissues from the different groups. Dotted lines indicated metastatic lesions. Magnification ×40 (upper panel) and ×100 (lower panel); scale bars: 200 μm (upper panel) and 100 μm (lower panel).

After considering these results, we further explored the role of KIAA1217 in HCC metastasis in vivo. Two groups of six nude mice each were injected intravenously in the tail vein with 1 × 10^6^ HepG2 cells that stably overexpressed KIAA1217 or the vector control. After eight weeks, the mice were sacrificed. The physical appearance, lungs, and spleens of the mice were photographed, and the tumor nodules on the surface of the lungs were counted. The mice injected with cells overexpressing KIAA1217 exhibited a more severe cachectic appearance than the vector group (Figure 3A). The spleens of the mice injected with cells overexpressing KIAA1217 were more enlarged than those of the vector group, potentially due to the stronger immune response in the mice injected with cells overexpressing KIAA1217 (Figure S3E). The number of nodules on the surface of the lungs in mice injected with cells overexpressing KIAA1217 was significantly increased compared to the vector group (Figure 3B). Subsequent H&E staining further confirmed that the nodules on the surface of the lungs were metastatic tumors (Figure 3C). Therefore, the in vitro and in vivo results indicate that KIAA1217 markedly promotes HCC cell migration and invasion and may play an important role in HCC metastasis.

### 2.3. KIAA1217 Induces EMT and Activates STAT3 in HCC Cells

**Figure 4:**
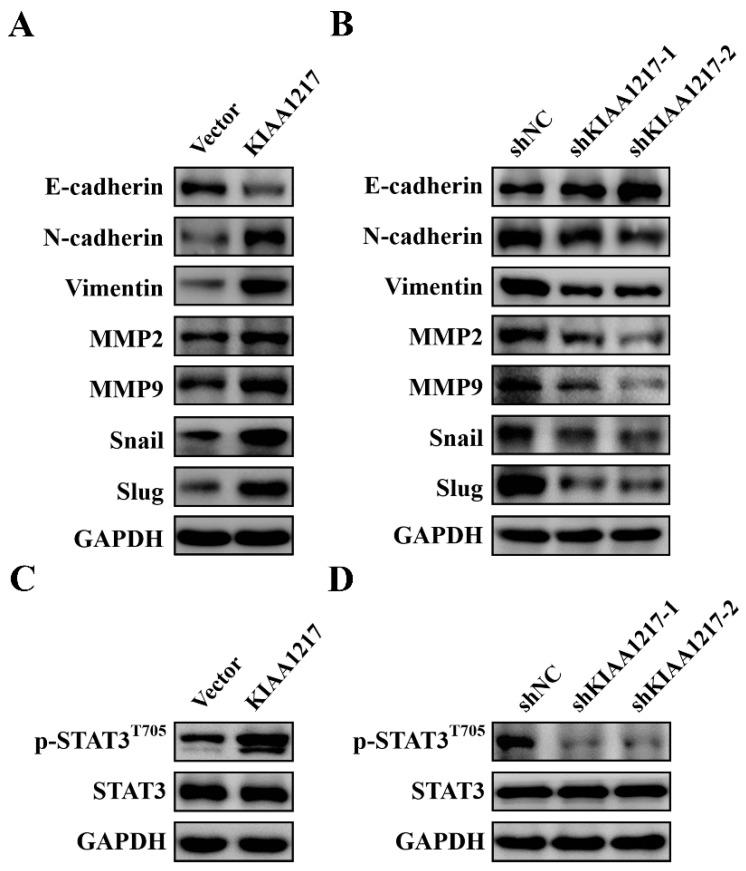
KIAA1217 induced EMT and activated STAT3 in HCC cells. (**A**,**B**) Western blot analysis of the expression of EMT markers in HepG2 cells with overexpression (**A**) or silencing of KIAA1217 (**B**). (**C**,**D**) Western blot analysis of the levels of phospho-STAT3 in HepG2 cells with overexpression (**C**) or knockdown of KIAA1217 (**D**). GAPDH was used as a loading control.

EMT plays a critical role in tumor invasion and metastasis, and thus we assessed the effect of KIAA1217 on EMT by analyzing the expression of EMT markers and EMT-related transcription factors. As shown in Figure 4A, Western blot analysis indicated significantly decreased expression of an epithelial marker (E-cadherin), increased expression of mesenchymal markers (N-cadherin and Vimentin) and matrix metalloproteinases (MMP2 and MMP9), and increased expression of EMT-related transcription factors (Snail and Slug) in HepG2 cells overexpressing KIAA1217. The opposite expression pattern of these proteins was observed in HepG2 cells with KIAA1217 silencing (Figure 4B). Based on these findings, KIAA1217 induces EMT to promote cell migration and invasion by activating Snail family expression in HCC cells.

EMT is precisely regulated by the signal transduction network involving multiple signaling pathways [23]. We tested the effects of KIAA1217 on EMT-related pathways to further investigate the potential mechanism underlying the induction of EMT by KIAA1217 in HCC cells. KIAA1217 significantly activated STAT3. As shown in Figure 4C, KIAA1217 overexpression in HepG2 cells markedly increased the levels of phosphorylated STAT3 (p-STAT3) compared with control cells. The opposite results were observed in HepG2 cells with KIAA1217 silencing (Figure 4D). The STAT3 signaling pathway has been reported to be involved in EMT induction and cancer metastasis in a variety of cancer types, including HCC [28,29]. Thus, we hypothesize that KIAA1217 may induce EMT by activating STAT3 in HCC cells.

### 2.4. KIAA1217 Induces EMT by Activating STAT3 in HCC Cells

**Figure 5:**
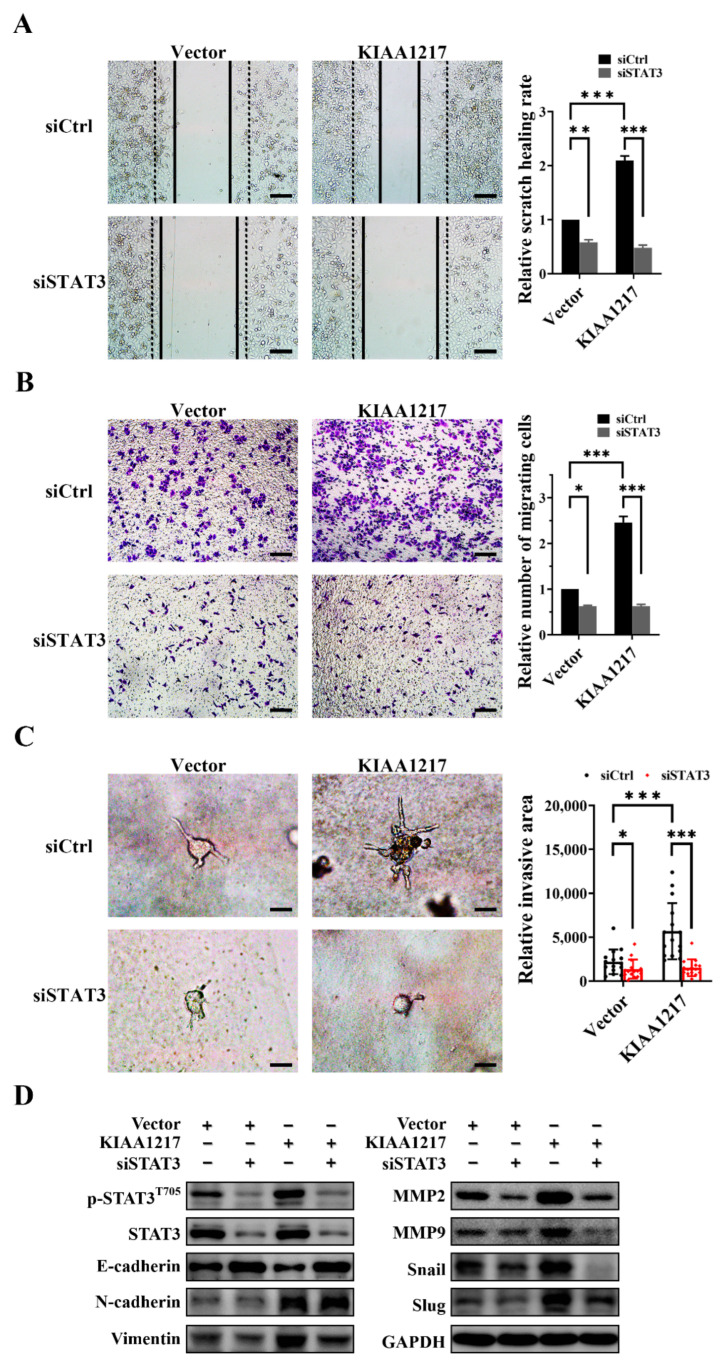
STAT3 knockdown abolished KIAA1217-mediated effects on increasing the migration, invasion, and EMT of HepG2 cells overexpressing KIAA1217. (**A**) The wound-healing assay was conducted at 48 h after scratching (magnification ×100; scale bars: 100 μm). (**B**) Transwell migration assay (magnification ×100; scale bars: 100 μm). (**C**) Cancer cell spheroid invasion assay in a 3D setting (magnification ×200; scale bars: 5 μm). siCtrl denotes the nontargeting control siRNA. (**D**) Western blot analysis of the expression of EMT markers. GAPDH served as a loading control. * *p* < 0.05; ** *p* < 0.01; *** *p* < 0.001. Error bars: SEM.

**Figure 6:**
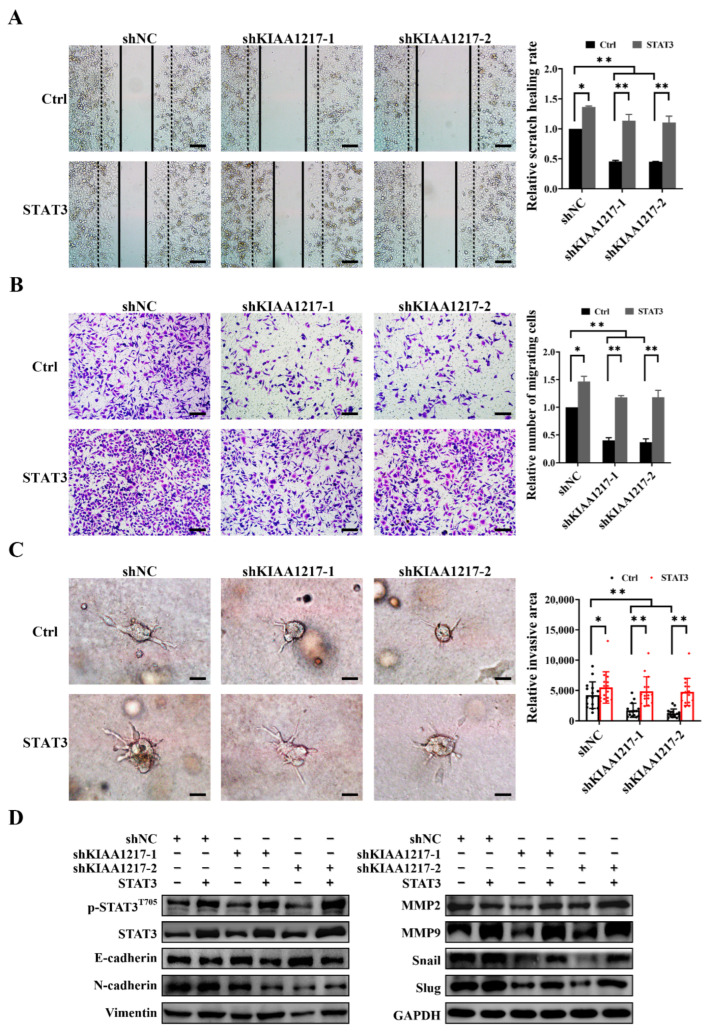
Overexpression of constitutively active STAT3 restored the impaired migration, invasion, and EMT of HepG2 cells with KIAA1217 knockdown. (**A**) The wound-healing assay was conducted at 48 h after scratching (magnification ×100; scale bars: 100 μm). (**B**) Transwell migration assay (magnification ×100; scale bars: 100 μm). (**C**) Cancer cell spheroid invasion assay in a 3D setting (magnification ×200; scale bars: 5 μm). Ctrl denotes control cells transfected with empty vectors. (**D**) Western blot analysis of the expression of EMT markers. GAPDH was used as a loading control. * *p* < 0.05; ** *p* < 0.01. Error bars: SEM.

We adopted two strategies to determine whether KIAA1217 induces EMT to promote HCC cell migration and invasion via STAT3 activation: downregulating STAT3 in HepG2 cells with KIAA1217 overexpression or activating STAT3 in HepG2 cells with KIAA1217 silencing. The siRNA targeting STAT3 was utilized to inhibit STAT3 in HepG2 cells overexpressing KIAA1217. Wound-healing and Transwell migration assays indicated that STAT3 knockdown significantly reduced the migration of HepG2 cells overexpressing KIAA1217 (Figure 5A,B). Similarly, a cancer cell spheroid invasion assay in a 3D setting showed that STAT3 silencing markedly decreased the invasion of HepG2 cells overexpressing KIAA1217 (Figure 5C). Conversely, constitutively active mutant STAT3 expression plasmids were transfected into cells to activate STAT3 in HepG2 cells with KIAA1217 knockdown. STAT3 overexpression substantially increased the migration and invasion of HepG2 cells with KIAA1217 silencing, as evidenced by the wound-healing assay (Figure 6A), transwell migration assay (Figure 6B), and cancer cell spheroid invasion assay in a 3D setting (Figure 6C). Furthermore, we also detected the expression of EMT markers and EMT-related transcription factors by performing Western blot analyses. As shown in Figure 5D, except for N-cadherin, STAT3 knockdown abrogated the KIAA1217-mediated increases in the expression of Vimentin, MMP2, MMP9, Snail, and Slug, as well as the decrease in E-cadherin expression in HepG2 cells overexpressing KIAA1217. Consistently, the overexpression of constitutively active STAT3 restored the expression levels of Vimentin, MMP2, MMP9, Snail, and Slug, but not N-cadherin, and decreased E-cadherin expression in HepG2 cells with KIAA1217 silencing (Figure 6D). Taken together, these data suggest that activation of STAT3 at least partially mediates the effects of KIAA1217 on promoting EMT and HCC cell migration and invasion.

### 2.5. KIAA1217 Interacts with STAT3 and JAK1/2 to Form a Complex in HCC Cells

Next, we may ask how KIAA1217 activates STAT3 in HCC cells and whether KIAA1217 and STAT3 interact to facilitate STAT3 activation. Therefore, we examined the interaction between KIAA1217 and STAT3/p-STAT3 through Co-IP analysis.

**Figure 7:**
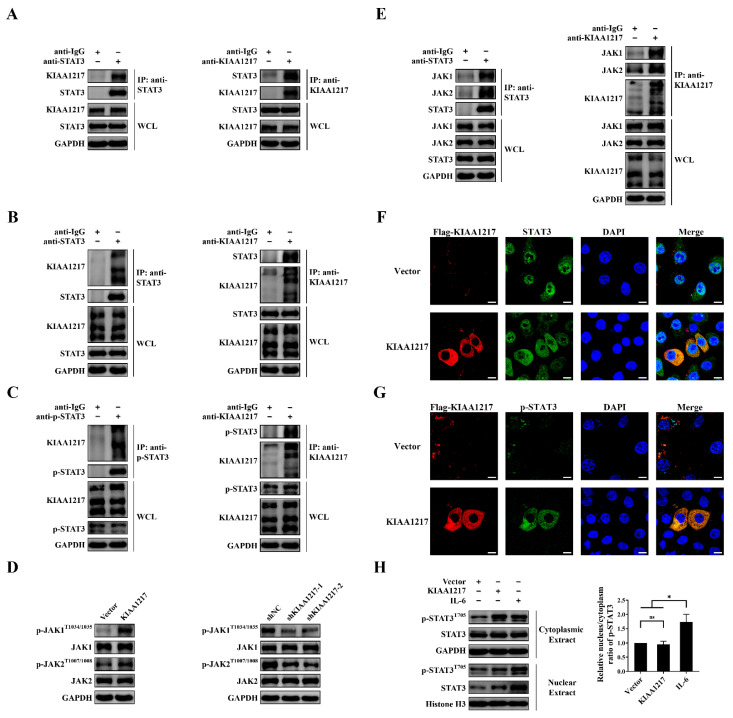
The interaction of KIAA1217 with STAT3/p-STAT3 and JAK1/2 in HCC cells. (**A**–**C**) Co-IP analysis of KIAA1217 and STAT3/p-STAT3 in HEK-293T cells ectopically expressing KIAA1217 and STAT3 (**A**) or in HepG2 cells (**B**,**C**). Isotype-matched IgG was used as a negative control. (**D**) Western blot analysis of the levels of JAK1/2 and p-JAK1/2 in HepG2 cells with KIAA1217 overexpression or knockdown. (**E**) Co-IP analysis of JAK1/2 with KIAA1217 or STAT3 in HepG2 cells. Isotype-matched IgG was used as a negative control. (**F**,**G**) Representative images of double IF staining for KIAA1217 (red) and STAT3 (**F**)/p-STAT3 (**G**) (green) captured with a laser scanning confocal microscope. The nucleus was stained with DAPI (blue). The assay was performed in HepG2 cells overexpressing KIAA1217. Magnification ×1000; scale bars: 10 μm. (**H**) Left panel, The cytoplasmic or nucleic STAT3/p-STAT3 levels in HepG2 cells overexpressing KIAA1217 or treated with IL-6 were detected using Western blot; right panel, quantitative evaluation of the Western blot results. ns: not significant; * *p* < 0.05. Error bars: SEM. GAPDH and Histone H3 were used as loading controls.

A reciprocal Co-IP analysis was first performed in HEK-293T cells ectopically expressing KIAA1217 and STAT3. As shown in Figure 7A, overexpressed KIAA1217 interacted with overexpressed STAT3, regardless of whether KIAA1217 or STAT3 was immunoprecipitated in HEK-293T cells. However, due to the low level of p-STAT3, the interaction of KIAA1217 and p-STAT3 was not detected. Then, we tested the interaction between endogenous KIAA1217 and STAT3/p-STAT3 in HepG2 cells. Again, the reciprocal Co-IP analysis in HepG2 cells revealed the binding between endogenous KIAA1217 and STAT3, regardless of which protein was immunoprecipitated (Figure 7B). Concomitantly, we also observed the interaction between endogenous KIAA1217 and p-STAT3 in HepG2 cells (Figure 7C).

Although we showed that KIAA1217 bound to and activated STAT3 in HCC cells, recent studies or functional domain predictions have not suggested that KIAA1217 possesses a kinase domain and functions as a kinase. Therefore, we speculate that JAKs, the classical upstream tyrosine kinases of STATs, may be recruited into the KIAA1217-STAT3 complex to execute the phosphorylation of STAT3 in HCC cells. First, we detected the effects of KIAA1217 on JAK1 or JAK2 activation. As shown in Figure 7D, Western blot analysis indicated increased or reduced levels of p-JAK1 and p-JAK2 in HepG2 cells following the overexpression or silencing of KIAA1217, respectively. Then, a Co-IP analysis was performed to detect the interaction of JAK1/2 with KIAA1217 or STAT3 in HepG2 cells. Endogenous JAK1 and JAK2 were present in endogenous KIAA1217- or STAT3-immunoprecipitated complexes and vice versa. (Figure 7E), indicating the interaction of KIAA1217 with STAT3 and JAK1/2 to form a complex in HepG2 cells.

In summary, KIAA1217 may function as an adaptor protein or scaffold protein to facilitate the interaction of STAT3 with JAK1 or JAK2 and subsequent activation of the JAK/STAT3 pathway in HCC cells.

### 2.6. p-STAT3 Activated by KIAA1217 Remains in the Cytoplasm Instead of Being Transported to the Nucleus

Commonly, activated STAT3 or p-STAT3 translocates from the cytoplasm into the nucleus to regulate target gene expression. Next, we conducted IF staining to observe the colocalization of KIAA1217 and STAT3 and the cellular localization of p-STAT3 activated by KIAA1217 in HepG2 cells transiently transfected with KIAA1217 expression plasmids. As shown in Figure 7F, KIAA1217 was localized in the cytoplasm of HepG2 cells transfected with the KIAA1217 plasmid, as described previously [5], and it was colocalized with STAT3 in the cytoplasm. Meanwhile, immunostaining for p-STAT3 showed that the fluorescence intensity of p-STAT3 was obviously increased in HepG2 cells overexpressing KIAA1217, while p-STAT3 was rarely detected in HepG2 cells without KIAA1217 overexpression or in vector control cells (Figure 7G), indicating that the p-STAT3 level was significantly increased when KIAA1217 was overexpressed in HepG2 cells, consistent with the results observed using Western blot (Figure 4C).

Importantly, we noticed that the elevated p-STAT3 level induced by KIAA1217 overexpression was primarily observed in the cytoplasm and was not localized in the nucleus as expected (Figure 7G). To exclude any occasional factors in our experimental environment that might have led to this phenomenon, IF staining was also performed in HepG2 cells treated with 5 ng/mL IL-6 for 30 min, as IL-6 is considered to be a classic cytokine that activates the STAT3 pathway. Most IL-6-induced p-STAT3 was transported to the nucleus rather than to the cytoplasm, and consistently, the nuclear STAT3 level was markedly increased (Figure S4), in marked contrast to the observation in HepG2 cells overexpressing KIAA1217 (Figure 7G). Furthermore, Western blot analysis was also performed to detect the cytoplasmic and nuclear p-STAT3 levels extracted from HepG2 cells with KIAA1217 overexpression or treated with IL-6. As shown in Figure 7H, the level of p-STAT3 in the nucleus was substantially increased in HepG2 cells treated with IL-6 compared with the cells overexpressing KIAA1217, and accordingly, the nuclear/cytoplasmic ratio of p-STAT3 was much higher in IL-6-treated HepG2 cells than in KIAA1217-overexpressing HepG2 cells. Taken together, these results confirm that KIAA1217-mediated STAT3 activation does not result in STAT3 nuclear translocation but instead maintains STAT3 in the cytoplasm.

### 2.7. Cytoplasmic p-STAT3 Mediates the Activation of Notch and Wnt/β-Catenin Pathways by KIAA1217 in HCC Cells

**Figure 8:**
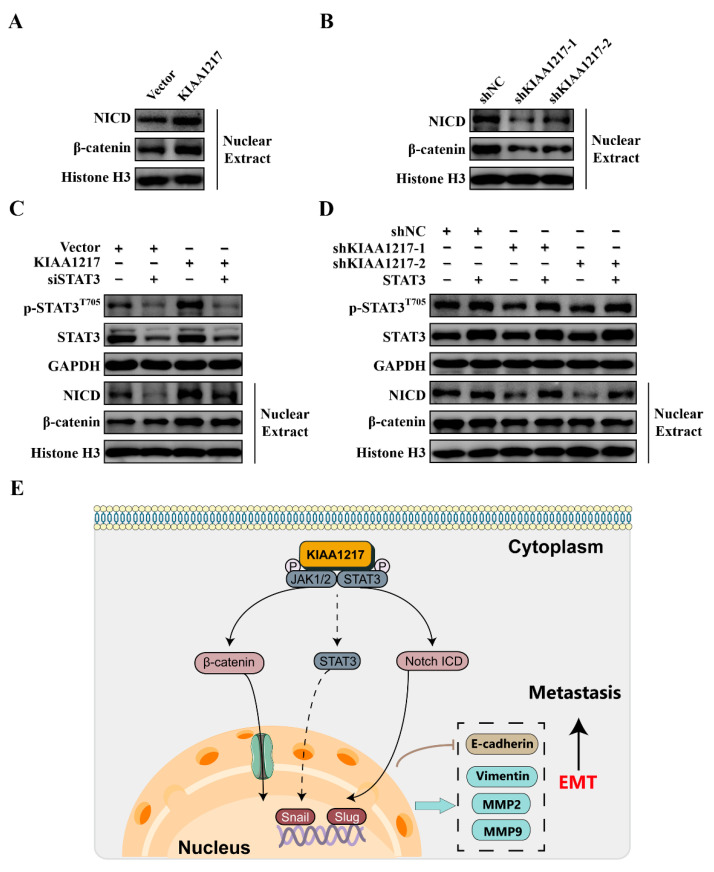
STAT3 mediates the activation of the Notch and Wnt/β-catenin pathways by KIAA1217 in HCC cells. Western blot analysis of the levels of nuclear NCID and β-catenin in HepG2 cells with KIAA1217 overexpression (**A**), KIAA1217 knockdown (**B**), KIAA1217 overexpression + STAT3 silencing (**C**), and KIAA1217 knockdown + constitutively active STAT3 expression (**D**). GAPDH and Histone H3 were used as loading controls for cytoplasmic and nuclear proteins, respectively. (**E**) Schematic diagram of the mechanism by which KIAA1217 regulates HCC metastasis. The dotted line indicates that only a small amount of p-STAT3 was transported to the nucleus.

In addition to the STAT3 pathway, we also tested the effects of KIAA1217 on other EMT-related pathways, such as the Notch and Wnt/β-catenin pathways. The Notch and Wnt/β-catenin pathways are well-known classical pathways that induce EMT. As shown in Figure 8A, KIAA1217 overexpression in HepG2 cells increased the levels of the Notch intracellular domain (NICD) and β-catenin in the nucleus, which indicated the activation of the Notch and Wnt/β-catenin pathways, respectively. Consistent with these findings, KIAA1217 knockdown decreased the levels of nuclear NICD and β-catenin in HepG2 cells (Figure 8B). Based on these results, KIAA1217 also mediated the activation of the Notch and Wnt/β-catenin pathways. Then, we speculated whether the Notch and Wnt/β-catenin pathways act downstream of cytoplasmic p-STAT3 activated by KIAA1217. Thus, we tested the effects of STAT3 activation or knockdown on nuclear NICD and β-catenin levels in HepG2 cells with KIAA1217 silencing or overexpression, respectively. Western blot analyses showed that STAT3 knockdown abolished the increase in nuclear NICD and β-catenin levels induced by KIAA1217 overexpression in HepG2 cells (Figure 8C). Conversely, the overexpression of constitutively active STAT3 restored the levels of nuclear NICD and β-catenin that were decreased due to KIAA1217 knockdown in HepG2 cells (Figure 8D). These data suggest that KIAA1217 activates STAT3 and maintains p-STAT3 in the cytoplasm, and then cytoplasmic p-STAT3 subsequently activates the Notch and Wnt/β-catenin pathways, which mediate the effects of KIAA1217 on promoting EMT and invasion and migration of HCC cells (Figure 8E).

## 3. Discussion

Although tumor invasion and metastasis are the main causes of a poor prognosis and cancer-related death, effective strategies that suppress cancer metastasis are still lacking in the clinic [30]. Therefore, analyses of the molecular drivers of tumor metastasis, such as aberrant actions of oncogenes or tumor suppressor genes, may help researchers identify therapeutic target genes to suppress metastasis. In this study, we showed for the first time that KIAA1217, a macromolecular protein with an unknown function, significantly promoted HCC metastasis by inducing EMT. We also revealed that high KIAA1217 expression was closely related to shorter survival of patients with HCC, indicating its potential as a therapeutic target for the suppression of HCC metastasis.

KIAA1217 is present in the HUGE protein database. The database was established by the Kazusa Gene Research Center in 1994 [4]. It is mainly used to analyze the proteins (>50 kD) encoded by newly recognized large cDNAs (>4 kb), and the genes encoding these proteins are uniformly named by KIAA plus four numbers [4]. To date, few reports have investigated KIAA1217, of which only a few studies have described its relation to the development of cancer [5,31,32,33,34]. Based on genetic association studies in pancreatic cancer databases, KIAA1217 is upregulated, suggesting that KIAA1217 may be a putative target for pancreatic cancer [32]. Copy number variation of KIAA1217 is also frequently observed in HCC [31] or breast cancer [34]. However, the potential role and mechanism of KIAA1217 in the progression of cancer remains unclear. In the present study, KIAA1217 was found to be frequently overexpressed in HCC cell lines and tissues. It is worth noting that KIAA1217 was low expressed in SMMC-7721 cells. We speculate that this is due to the extraordinary heterogeneity of HCC [14]. Furthermore, overexpression and knockdown experiments indicated that KIAA1217 promoted cell migration and invasion by inducing EMT in vitro. Nevertheless, it had no effect on HCC proliferation. Consistently, a metastasis assay in vivo showed that the mice intravenously injected with HCC cells overexpressing KIAA1217 exhibited more severe cachexia and a greater number of HCC nodules on the surface of the lungs, indicating that KIAA1217 significantly enhanced lung metastasis in vivo. Clinically, patients with HCC presenting high KIAA1217 expression experienced shorter OS and DFS times than patients with low expression. In addition, in contrast to the matched nontumor tissues, KIAA1217 expression was increased in the 18 other types of cancers, which was analyzed using the GEPIA web server [25] (Figure S5). This finding suggests that KIAA1217 may also play a certain role in other tumors, which deserves further investigation. Taken together, our study shows that KIAA1217 may function as an oncogene and promote HCC invasion and metastasis in vitro and in vivo.

In addition, a novel KIAA1217-RET fusion gene was identified in lung adenocarcinomas and revealed that the KIAA1217-RET fusion gene functions as an oncogenic driver gene [5]. Interestingly, another study by our team also observed a truncated transcript of KIAA1217 expressed in ovarian cancer cell lines (unpublished data). This finding suggests that a fusion gene consisting of part of the KIAA1217 sequence and another gene exists in ovarian cancer; however, this requires further validation. The occurrence of genomic instability, including gene fusion or rearrangement, is one of the important mechanisms inducing cancer [35]. Therefore, KIAA1217, an oncogene, also appears to participate in the occurrence and development of cancer in the form of fusion genes via genome rearrangements.

Mechanistic studies revealed that KIAA1217 induced EMT by interacting with and activating STAT3 and that KIAA1217 mediated the activation of STAT3 by recruiting and activating JAK1/2. The STAT3 pathway is a classic signaling pathway in cells that is also closely related to the progression of cancer [28,36]. Our study further confirmed an essential role for the STAT3 pathway in EMT and metastasis of HCC. Notably, the KIAA1217-induced expression of N-cadherin, an EMT-related marker, did not appear to be affected by STAT3 to the same extent as the expression of other markers. Therefore, we considered that other pathways might also be involved in KIAA1217-induced expression of N-cadherin. Interestingly, the interaction of KIAA1217 and p-STAT3 prevented activated p-STAT3 from being transported to the nucleus, as most p-STAT3 was retained in the cytoplasm. Activated p-STAT3 is widely considered functional by the activation of transcription after transport to the nucleus [28]; however, our study suggested a different functional pattern of STAT3, in which most p-STAT3 remained in the cytoplasm and coordinated the Notch and Wnt/β-catenin pathways to facilitate EMT induction and HCC metastasis triggered by KIAA1217. Considering the structural features of the KIAA1217 protein predicted by the Pfam website and its ubiquitous expression in the cytoplasm, we speculate that KIAA1217 may function as an adaptor protein or scaffold protein to regulate the signaling pathway network.

Notably, we occasionally observed that higher expression of KIAA1217 was significantly more common in cirrhotic liver tissues than in normal liver, adjacent nontumor, and HCC tissues when performing IHC analyses of the TMA slide to compare the KIAA1217 expression levels in adjacent nontumor and HCC tissues (Figure 1C). Liver cirrhosis is the final pathological result of liver damage resulting from various chronic liver diseases and is characterized by tissue fibrosis and the conversion of the normal liver architecture into structurally abnormal nodules [37]. Liver cirrhosis is also a major risk factor for the development of HCC, and almost all patients with HCC are preceded by cirrhosis [27]. Although liver cirrhosis is caused by many factors, liver fibrosis, which is the precursor of cirrhosis, is a key pathological process of all chronic liver diseases evolving into cirrhosis [37,38,39]. Myofibroblasts are a critical cell type involved in liver fibrosis [38]. According to recent studies, the transdifferentiation of hepatic stellate cells is the major source of myofibroblasts [24,26,37,39]. Moreover, accumulating evidence indicates that myofibroblasts are also derived from portal fibroblasts, circulating fibrocytes, bone marrow, and EMT of hepatocytes and cholangiocytes [38,39]. Since we documented that KIAA1217 induced EMT in HCC cells and was highly expressed in cirrhotic liver tissues, we speculate that KIAA1217 may play a certain role in the progression of liver fibrosis and liver cirrhosis, which requires further investigation. Moreover, further studies are needed to determine whether KIAA1217 represents a potential biomarker for the prediction of disease progression or a therapeutic target in cirrhosis in the future.

In conclusion, KIAA1217, which may function as an adaptor protein or scaffold protein in the cytoplasm, forms a complex with JAK1/2 and STAT3 and facilitates STAT3 activation. Then, activated STAT3 remains in the cytoplasm to further activate the Notch and Wnt/β-catenin pathways, through which KIAA1217 induces EMT and accordingly is involved in promoting HCC metastasis (Figure 8E). Therefore, KIAA1217 is identified as an oncogenic protein during HCC progression and may serve as a putative antimetastatic target for HCC treatment.

## 4. Materials and Methods

### 4.1. Cell Culture

Four HCC cell lines (HepG2, Huh7, SMMC-7721, and BEL-7402), the human normal hepatocyte cell line L02, and the human embryonic kidney cell line HEK-293T were purchased from the Cell Bank of the Chinese Academy of Sciences (Shanghai, China). HEK-293T cells were cultured in Dulbecco’s modified Eagle’s medium (DMEM, 10-013-CVRC, Corning, Suzhou, Jiangsu, China) supplemented with 10% fetal bovine serum (FBS, VS500T, Ausbian, Sydney, Australia) and a 1% penicillin-streptomycin solution (15070063, Gibco, Waltham, MA, USA), and other cell lines were maintained in RPMI-1640 medium (10-040-CVRC, Corning, Suzhou, Jiangsu, China) supplemented with 10% FBS and a 1% penicillin-streptomycin solution. All cell lines were cultured at 37 °C with 5% CO_2_ in an incubator.

### 4.2. Tissue Microarray (TMA)

HCC TMA slide (HlivH060CD03) was purchased from Outdo Biotechnology Co., Ltd. (Shanghai, China). All patients provided consent for the use of their tissue samples and clinical data. This study was approved by the Committees for Ethical Review of Research at Northeast Normal University.

### 4.3. Animals

Female athymic BALB/c nude mice (4–6 weeks old) were purchased from Changsheng Biotechnology Co., Ltd. (Benxi, Liaoning, China) and raised under specific pathogen-free conditions. Animal care and experimental protocols were conducted with approval from the Northeast Normal University Experimental Animal Care Commission of China and performed following established guidelines.

### 4.4. Plasmids and siRNA Transfection

The KIAA1217 expression plasmid was constructed and synthesized by GenScript Biotechnology Co., Ltd. (Nanjing, Jiangsu, China), in which the full-length ORF of the human KIAA1217 gene (NM_019590.5) was cloned into the pcDNA3.1-DYK vector. The pCMV-DYK-STAT3 expression plasmid was constructed and maintained in our laboratory. The empty vector, named Vector, served as the negative control.

pGreenPuro lentiviral vectors containing short hairpin RNAs (shRNAs) targeting KIAA1217 were constructed and synthesized by GENEWIZ Biotechnology Co., Ltd. (Suzhou, Jiangsu, China) and designated shKIAA1217-1 and shKIAA1217-2. The lentiviral vector containing the nontargeting shRNA named shNC was used as the negative control. The designed target sequences are described in Supplementary Table S1.

The siRNA targeting STAT3 was designed and synthesized by GenePharma (Suzhou, Jiangsu, China). The sequences of the siRNAs are listed in Supplementary Table S1.

Lipofectamine 2000 Transfection Reagent (11668019, Thermo Fisher Scientific, Waltham, MA, USA) or X-tremeGENE HP DNA Transfection Reagent (6366236001, Roche, Mannheim, Germany) was used for plasmid transfection. Lipofectamine 2000 Transfection Reagent or X-tremeGENE siRNA Transfection Reagent (4476093001, Roche, Mannheim, Germany) was used for siRNA transfection. Transfections were performed according to the manufacturer’s protocols.

### 4.5. Establishment of Stable Cell Lines

The HepG2 cell line stably overexpressing KIAA1217 was obtained by selection for G418 (10131027, Gibco, Waltham, MA, USA) resistance. KIAA1217, or empty expression plasmids, were transfected into HepG2 cells using Lipofectamine 2000 Transfection Reagent according to the manufacturer’s protocols, and the transfected cells were subsequently selected with G418 (200 μg/mL) for 2 weeks to generate stable cell lines. The stably transfected cells were validated by performing RT–qPCR and Western blot analyses. The stable cell lines were then maintained in culture medium supplemented with 200 μg/mL G418.

Stable KIAA1217 knockdown in HepG2 cells was generated by lentivirus-mediated shRNA transduction. For lentiviral packaging, HEK-293T cells were cotransfected with psPAX2, pMD2.G, and pGreenPuro (shKIAA1217-1, shKIAA1217-2, or shNC) using Lipofectamine 2000 Transfection Reagent according to the manufacturer’s protocols. 48 h after transfection, the viral supernatant was collected. HepG2 cells were incubated with virus-containing supernatant for 12–16 h in the presence of 10 μg/mL polybrene (C0351, Beyotime, Shanghai, China). The infected cells were then selected with puromycin (2 μg/mL) (ST551, Beyotime, Shanghai, China) for 48 h. Stable knockdown cells were confirmed using RT–qPCR and Western blot analyses.

### 4.6. RNA Isolation and Real-Time Quantitative PCR (RT–qPCR)

Total RNA was isolated using TRIzol reagent (15596026, Invitrogen, Waltham, MA, USA) and reverse-transcribed into cDNAs with EasyScript^®^ First-Strand cDNA Synthesis SuperMix (AE301-02, TransGen Biotech, Beijing, China) according to the manufacturer’s protocols.

The cDNA templates were then subjected to RT–qPCR using the SYBR Green PCR Kit (04913914001, Roche, Mannheim, Germany) according to the manufacturer’s instructions. The assay was performed with a PikoReal 96 PCR System (Thermo Fisher Scientific, Waltham, MA, USA). β-Actin was used as an internal control. The relative expression levels were quantified and analyzed using PikoReal 2.1 software (Thermo Fisher Scientific, Waltham, MA, USA). The relative expression levels of the target genes were determined using the 2^−ΔΔCt^ method. Each sample was analyzed in triplicate. The primers were all synthesized and purchased from GENEWIZ Biotechnology Co., Ltd. (Suzhou, Jiangsu, China). The primer sequences are listed in Supplementary Table S2.

### 4.7. Western Blot Assay

Total proteins and nuclear/cytoplasmic proteins were extracted with RIPA lysis buffer (P0013B, Beyotime, Shanghai, China) and a Nuclear-Cytoplasmic Extraction Kit (P0028, Beyotime, Shanghai, China), respectively. Protein lysates were separated by sodium dodecyl sulfate-polyacrylamide gel electrophoresis (SDS–PAGE) and transferred onto polyvinylidene fluoride (PVDF) membranes (88518, Thermo Fisher Scientific, Waltham, MA, USA). The membranes were blocked with TBST (ST673, Beyotime, Shanghai, China) containing 5% skim milk (P0216, Beyotime, Shanghai, China) for 1 hr at room temperature and then incubated with appropriate antibodies at 4 °C overnight. After washing, the membranes were incubated with HRP-conjugated anti-IgG (Proteintech, Wuhan, Hubei, China) for 2 h at room temperature. The antigen-antibody complexes on the membranes were detected with High-sig ECL reagent (180-5001, Tanon, Shanghai, China). The images were acquired using the MicroChemi system (70-25-00, NDR Bio-Imaging Systems, Jerusalem, Israel) and quantified with ImageJ software (ImageJ 1.50i, National Institutes of Health, Bethesda, MD, USA). GAPDH and Histone H3 served as internal controls. The antibodies used in this study are listed in Supplementary Table S3.

### 4.8. Wound-Healing Assay

The wound-healing assay was used to evaluate cell migration. Transfected cells were cultured on 6-well plates until reaching confluence, then a line was scraped in the cellular monolayer using sterile 200 μL pipette tips, and then the cells were cultured in a serum-free medium. Images of cell migration toward the wound were captured under an inverted microscope (BX50, Olympus, Tokyo, Japan) at 0, 24, 48, and 72 h after scratching. The migration area was quantified using ImageJ software to assess the rate of wound closure.

### 4.9. Transwell Migration Assay

The cell migration capacity was also assessed using the Transwell migration assay. Transwell chambers (3422, Corning, New York, NY, USA) with an 8 μm pore size polycarbonate membrane were used to perform the 2-chamber migration assay. Transfected cells (5 × 10^4^) suspended in 200 μL of serum-free medium were seeded into the upper chambers of 24-well plates, and 600 μL of medium containing 10% FBS were added into the lower chambers as the attractant. After incubation at 37 °C for 48 h, non migrated cells that remained on the upper surface of the membranes were removed with cotton swabs, and the cells that had migrated to the lower surface of the membranes were fixed with 4% paraformaldehyde (P0099, Beyotime, Shanghai, China) and stained with 0.1% crystal violet (C0121, Beyotime, Shanghai, China). Ultimately, the migrated cells were captured and counted in five random fields per well under an inverted microscope to assess the cell migration rate.

### 4.10. Cancer Cell Spheroid Invasion Assay in a Three-Dimensional (3D) Setting

Tumor cell invasion was assessed using a 3D cancer cell spheroid invasion assay [40]. The cells inoculated on the lid of 10 cm dishes were cultured in hanging drops for 72 h to form spheroids (approximately 1 × 10^3^ cells per droplet). The spheroids were collected from the lid and mixed with Matrigel (356234, Corning, New York, NY, USA) and collagen type I (354236, Corning, New York, NY, USA). Then, the polymers were embedded in 24-well plates for 30 min at 37 °C to generate 3D culture systems, followed by submergence of the 3D cultures in the cell culture medium. After 48 h, images of invading cells were captured using an inverted microscope. The invasion area was quantified using ImageJ software to assess the invasion capacity of tumor cells.

### 4.11. Immunofluorescence (IF) Staining Assay

Cells were seeded onto coverslips in 24-well plates and then fixed with 4% paraformaldehyde, permeabilized with 0.2% Triton X-100 (ST797, Beyotime, Shanghai, China), and blocked with 5% bovine serum albumin (BSA, A8020, Solarbio, Beijing, China). After incubation with appropriate antibodies at 4 °C overnight, cells were incubated with Cy3 or FITC-labeled anti-IgG (Beyotime, Shanghai, China) at room temperature, followed by nuclear counterstaining with DAPI (C1005, Beyotime, Shanghai, China). Images were captured using a laser scanning confocal microscope (LSM 880, ZEISS, Oberkochen, Germany). The antibodies used in this study are shown in Supplementary Table S3.

### 4.12. Immunohistochemistry (IHC) Assay

An immunohistochemistry assay was performed to detect KIAA1217 expression in the TMA tissue samples. The TMA slide was dewaxed in xylene and rehydrated with a series of graded alcohol solutions. Then, antigen retrieval was performed with sodium citrate buffer (C1032, Solarbio, Beijing, China). According to the protocols of the two-step detection kit (PV-9001, ZSGB-BIO, Beijing, China), the TMA slide was incubated in an appropriate endogenous peroxidase blocker for 10 min at room temperature, followed by incubation with anti-SKT (KIAA1217 is also known as SKT) antibodies overnight at 4 °C and subsequent incubation with response enhancer and enhanced enzyme-labeled goat anti-rabbit IgG polymer for 20 min at room temperature. A DAB Chromogenic Kit (ZLI-9017, ZSGB-BIO, Beijing, China) was used to detect antibody binding, and the reaction was stopped by immersing the TMAs in running water once a brown color appeared. Finally, the TMA slide was counterstained with hematoxylin (G1121, Solarbio, Beijing, China), dehydrated using a series of graded alcohol solutions, and mounted. Images were photographed with an inverted microscope. Appropriate positive and negative controls were included for each run of the IHC assay. The antibodies used in this study are listed in Supplementary Table S3.

### 4.13. Coimmunoprecipitation (Co-IP) Assay

Co-IP was performed using Protein A/G Magnetic Beads (HY-K0202, MedChemExpress, Monmouth Junction, NJ, USA) according to the manufacturer’s protocols to confirm protein-protein interactions. 25 μL of magnetic beads pretreated with PBST (0.5% Triton X-100 in PBS) solution were fully suspended with PBST containing the antibodies at a final concentration of 5 μg/mL and incubated at 4 °C for 2 h in a flip mixer. Proteins extracted from cells using IP lysis solution (87787, Thermo Fisher Scientific, Waltham, MA, USA) were fully suspended with the antibody-magnetic bead complexes and incubated at 4 °C overnight in a flip mixer (88881002, Thermo Fisher Scientific, Waltham, MA, USA). After thorough washing, the antigen-antibody-magnetic bead complexes were boiled in 25 μL of 1 × SDS–PAGE loading buffer, and the supernatant was subjected to Western blot analysis.

### 4.14. Metastasis Assay In Vivo

Female athymic BALB/c nude mice (4–6 weeks old) were used to establish the metastasis model in vivo. HepG2 cells (1 × 10^6^) stably overexpressing KIAA1217 or the control vector were injected into the tail vein of the mice. After 8 weeks, the mice were sacrificed, and the physical appearance, lungs, and spleens of the mice were photographed. Then, the tumor nodules on the surface of the lungs were separately counted by two researchers. Subsequently, the lungs were excised, fixed with a neutral formalin solution, and embedded in paraffin. The tissues were then serially sectioned and stained with hematoxylin and eosin (H&E) to identify the pathological characteristics. H&E staining was performed using an improved H&E staining kit (G1121, Solarbio, Beijing, China) according to the manufacturer’s protocols.

### 4.15. Statistical Analysis

Statistical analyses were conducted using SPSS 21.0 software or GraphPad Prism 8 software. The data are presented as the means ± standard errors of the means (SEM) from at least three independent experiments. The differences between groups were analyzed with Student’s *t*-test when only two groups were compared or using one-way analysis of variance (ANOVA) or two-way ANOVA when more than two groups were compared. Overall survival and disease-free survival curves were obtained using Kaplan–Meier analysis, and differences were compared with the log-rank test. A two-tailed *p*-value < 0.05 was considered statistically significant.

## Data Availability

The data presented in this study are available on request from the corresponding author.

## Supplementary Materials

The following are available online at https://www.mdpi.com/article/10.3390/ijms23010104/s1.
