# Supplementary material for: KIAA1217 Promotes Epithelial-Mesenchymal Transition and Hepatocellular Carcinoma Metastasis by Interacting with and Activating STAT3
Source: Int J Mol Sci. 2021 Dec 22;23(1):104. doi: 10.3390/ijms23010104 (PMC8745027; doi:10.3390/ijms23010104)
Supplement: Supplementary file 1 [file ijms-23-00104-s001.zip › ijms-1503219-supplementary.pdf]

Supplementary Figures

Name	Exon	CDS (bp)	Amino acid (aa)	Protein (kD)
KIAA1217-V1	21	5832	1943	214
KIAA1217-V2	19	3795	1264	138
KIAA1217-V4	19	4125	1374	150
KIAA1217-V5	18	4050	1349	147
KIAA1217-V6	14	2799	932	103
KIAA1217-V7	12	2601	866	95
KIAA1217-V8	13	3186	1061	121

Supplementary Figure S1. Information of various KIAA1217 splicing variants according to the NCBI database.

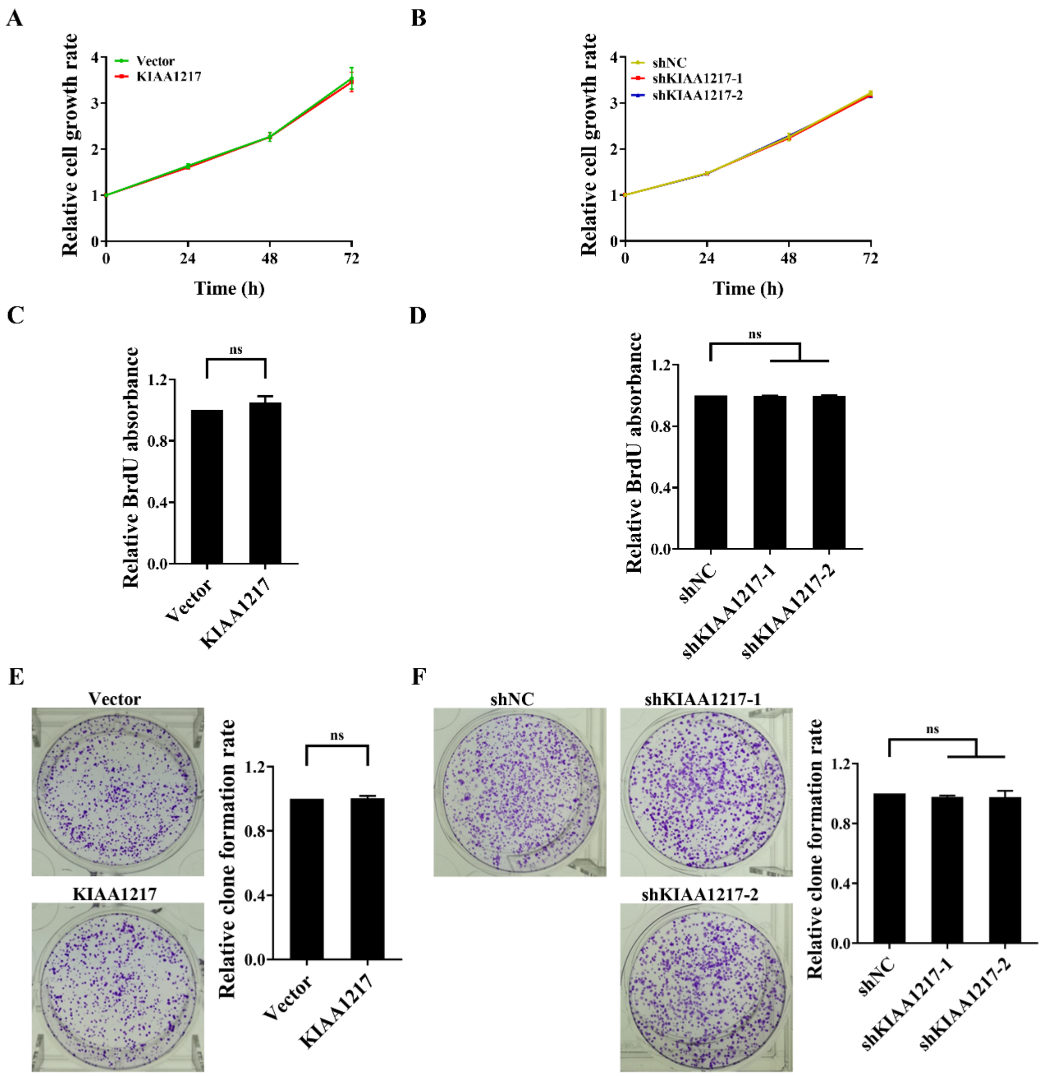

Supplementary Figure S2. KIAA1217 was not involved in regulating HCC proliferation. (A and B) MTT assays were performed to detect the proliferation of HepG2 cells with KIAA1217 overexpression (A) or knockdown (B). (C and D) BrdU incorporation assays were performed to detect the proliferation of HepG2 cells with KIAA1217 overexpression (C) or silencing (D). (E and F) Colony formation assays were performed to detect the proliferation of HepG2 cells with KIAA1217 overexpression (E) or knockdown (F). ns, not significant. Error bars, SEM.

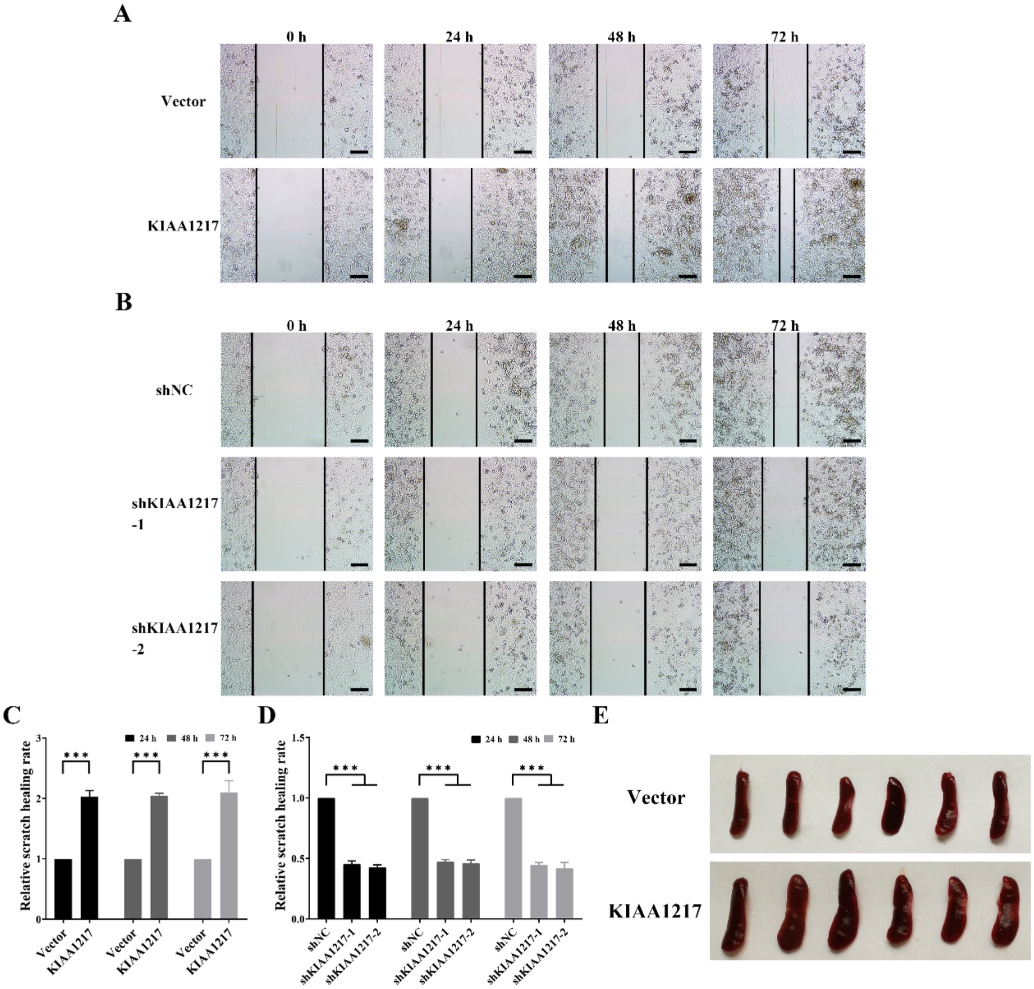

Supplementary Figure S3. KIAA1217 promoted cell migration and invasion *in vitro* and lung metastasis *in vivo*. (A-D) The wound-healing assay was performed to detect cell migration in HepG2 cells with KIAA1217 overexpression (A) and KIAA1217 silencing (B) at 0, 24, 48, and 72 hrs after scratch. Magnification, × 100; scale bars, 100 μm. (C) Measurement and statistical analysis of A. (D) Measurement and statistical analysis of B. ***P < 0.001. Error bars, SEM. (E) Representative images of spleens in each group of nude mice in lung metastasis experiment *in vivo*.

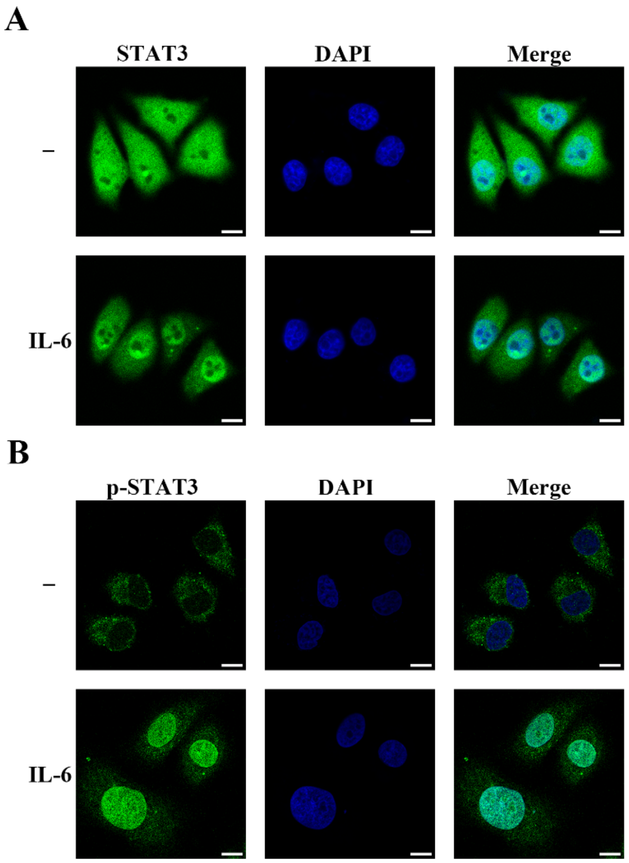

Supplementary Figure S4. Immunofluorescence analysis of STAT3/p-STAT3 in HCC cells treated with IL-6. HepG2 cells were treated with IL-6 under 5 ng/mL for 30 mins. Representative images of IF staining for STAT3 (A) and p-STAT3 (B) (green) by a laser scanning confocal microscope. The nucleus was stained with DAPI (blue). Magnification, × 1000; scale bars, 10 μm.

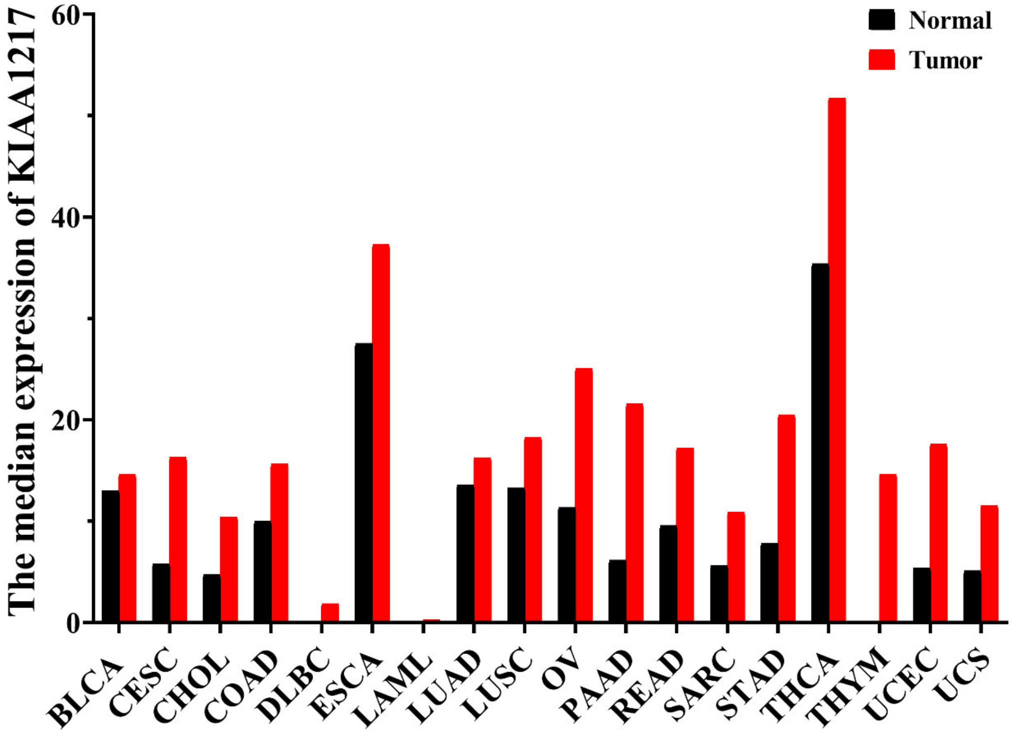

Supplementary Figure S5. KIAA1217 expression profile across 18 types of cancer samples and paired normal tissues from the GEPIA web server [1]. BLCA, Bladder Urothelial Carcinoma; CESC, Cervical squamous cell carcinoma and endocervical adenocarcinoma; CHOL, Cholangio carcinoma; COAD, Colon adenocarcinoma; DLBC, Lymphoid Neoplasm Diffuse Large B-cell Lymphoma; ESCA, Esophageal carcinoma; LAML, Acute Myeloid Leukemia; LUAD, Lung adenocarcinoma; LUSC, Lung squamous cell carcinoma; OV, Ovarian serous cystadenocarcinoma; PAAD, Pancreatic adenocarcinoma; READ, Rectum adenocarcinoma; SARC, Sarcoma; STAD, Stomach adenocarcinoma; THCA, Thyroid carcinoma; THYM, Thymoma; UCEC, Uterine Corpus Endometrial Carcinoma; UCS, Uterine Carcinosarcoma.

Supplementary Tables

Supplementary Table S1. The sequences of shRNAs and siRNAs used in this study

Name	Sequence
shKIAA1217-1	GGAACGCCTTTCTAATGGA
shKIAA1217-2	GGAAATGCATATGGAACAA
shNC	TTCTCCGAACGTGTCACGT
siSTAT3	Sense: CCACUUUGGUGUUUCAUAATT<br>Antisense: UUAUGAAACACCAAAGUGGTT
siNC	Sense: UUCUCCGAACGUGUCACGUTT<br>Antisense: ACGUGACACGUUCGGAGAATT

Supplementary Table S2. The sequences of RT-qPCR primers used in this study

Target gene	Sequence	Amplification length (bp)
KIAA1217	F: GCGATGCGAAGTCGGAAGT	277
	R: GCCCTGTTCTTGGCACTCAC	
β-actin	F: CGTGCGTGACATTAAGGAGAAG	176
	R: GGAAGGAAGGCTGGAAGAGTG	

Supplementary Table S3. List of the antibodies used in this study

Name	Catalog number	Source
KIAA1217	24880-1-AP	Proteintech
SKT	ab121223	Abcam
Flag	F1804	Sigma
E-cadherin	20874-1-AP	Proteintech
N-cadherin	22018-1-AP	Proteintech
Vimentin	10366-1-AP	Proteintech
MMP2	sc-13594	Santa Cruz
MMP9	10375-2-AP	Proteintech
Snail	3879	Cell Signaling Technology
Slug	12129-1-AP	Proteintech
STAT3	sc-8019	Santa Cruz
phospho-STAT3	9145	Cell Signaling Technology
AKT	60203-2-Ig	Proteintech
phospho-AKT	5012	Cell Signaling Technology
p44/42 MAPK (ERK1/2)	4695	Cell Signaling Technology
phospho-p44/42 MAPK (ERK1/2)	4370	Cell Signaling Technology
β-catenin	51067-2-AP	Proteintech
Cleaved Notch 1	4147	Cell Signaling Technology
JAK1	3344	Cell Signaling Technology
phospho-JAK1	3331	Cell Signaling Technology

JAK2	3230	Cell Signaling Technology
phospho-JAK2	3771	Cell Signaling Technology
GAPDH	60004-1-Ig	Proteintech
Histone H3	17168-1-AP	Proteintech

References

1. Berens, E.B.; Holy, J.M.; Riegel, A.T.; Wellstein, A. A cancer cell spheroid assay to assess invasion in a 3D setting. *J Vis Exp* **2015**, *105*, e53409-e53414.
